# Detection and mitigation of DDoS attacks based on multi-dimensional characteristics in SDN

**DOI:** 10.1038/s41598-024-66907-z

**Published:** 2024-07-16

**Authors:** Kun Wang, Yu Fu, Xueyuan Duan, Taotao Liu

**Affiliations:** 1 Department of Information Security, Naval University of Engineering, Wuhan, 430033 China; 2 School of Mathematics and Information Engineering, Xinyang Vocational and Technical College, Xinyang, 464000 China; 3 College of Computer and Information Technology, Xinyang Normal University, Xinyang, 464000 China; 4 Henan Key Laboratory of Analysis and Applications of Education Big Data, Xinyang, 464000 China

**Keywords:** Deep learning, Software defined network, Distributed denial of service, Attack detection, Computer science, Information technology, Software

## Abstract

Due to the large computational overhead, underutilization of features, and high bandwidth consumption in traditional SDN environments for DDoS attack detection and mitigation methods, this paper proposes a two-stage detection and mitigation method for DDoS attacks in SDN based on multi-dimensional characteristics. Firstly, an analysis of the traffic statistics from the SDN switch ports is performed, which aids in conducting a coarse-grained detection of DDoS attacks within the network. Subsequently, a Multi-Dimensional Deep Convolutional Classifier (MDDCC) is constructed using wavelet decomposition and convolutional neural networks to extract multi-dimensional characteristics from the traffic data passing through suspicious switches. Based on these extracted multi-dimensional characteristics, a simple classifier can be employed to accurately detect attack samples. Finally, by integrating graph theory with restrictive strategies, the source of attacks in SDN networks can be effectively traced and isolated. The experimental results indicate that the proposed method, which utilizes a minimal amount of statistical information, can quickly and accurately detect attacks within the SDN network. It demonstrates superior accuracy and generalization capabilities compared to traditional detection methods, especially when tested on both simulated and public datasets. Furthermore, by isolating the affected nodes, the method effectively mitigates the impact of the attacks, ensuring the normal transmission of legitimate traffic during network attacks. This approach not only enhances the detection capabilities but also provides a robust mechanism for containing the spread of cyber threats, thereby safeguarding the integrity and performance of the network.

## Introduction

Software Defined Network (SDN), by decoupling the network's logical control and data forwarding functions, enables the centralized and flexible configuration of network forwarding rules, allowing the network to evolve independently of hardware^1^^.^ With its open interface model, programmable forwarding policies, and scalable network size, it is gradually becoming a popular network architecture in cloud computing and big data environments^2^.

In fact, at the beginning of SDN design, it mainly focused on how to schedule and allocate network resources, so that people could realize automatic control of network services through software, but there were not many considerations about its security. Taking the mainstream OpenFlow protocol as an example, when a new flow (one that has no matching rules in the switch flow table) arrives at the switch, the switch will send a Packet-in packet to the upper controller to ask about the processing method of the flow. The controller receives the request message, analyzes the forwarding request, calculates the forwarding path, and sends the flow processing strategy to the switch it is connected to. The switch updates its flow table according to the received policy information and then processes all subsequent actions according to the rules in the flow table without consulting the controller again. This open network architecture can dynamically allocate network resources and improve the efficiency of network link utilization. However, the openness of SDN also makes it more susceptible to cyber-attacks. For instance, flood attacks launched by malicious actors can lead to the exhaustion of the controller's computational resources, congestion of shared links, and overflow of the switch's buffer area. Additionally, attackers could exploit the open API interfaces to steal or tamper with non-public data within the system, resulting in the leakage or loss of critical system information. Among various attacks targeting SDN, Distributed Denial of Service (DDoS) attacks are a common, easily organized, and highly impactful type of cyber attack. Attackers typically use forged IP addresses or control a large number of zombie hosts to continuously send attack packets from any terminal connected to the forwarding device, causing the switch or controller to become overloaded and unable to respond to normal network service requests promptly. This can lead to a degradation or even paralysis of the SDN network's service quality^3^. Therefore, the detection of DDoS attacks and the mitigation of their effects are gradually becoming a hot issue in the field of SDN application research.

DDoS attack detection in an SDN environment refers to the use of certain technical means to inspect and analyze traffic data within the SDN to uncover potential attack behaviors within the network. Traditional detection methods include: Statistical-based methods^4^, Information theory-based methods^5^, Clustering-based methods^6^, Machine learning-based methods^7–9^. However, these methods generally face several issues:

- Redundancy in data features, which complicates the analysis process.
- High computational overhead, as the models require significant processing power and time to analyze data.
- Insufficient extraction of feature information, leading to suboptimal detection accuracy.
- The need for improved accuracy in detection methods.

Attack mitigation primarily involves using certain means or strategies to reduce the impact and damage of DDoS attacks on SDN networks. There are typically two methods of implementation: One is to reduce the entry of attack traffic into the network, mitigating the shock effect of DDoS attacks on the network. The other is to divert the traffic from the network devices under attack to devices with lighter loads, ensuring the overall service quality of the SDN does not significantly deteriorate through load balancing. However, neither of these methods can eliminate the impact of DDoS attacks on SDN networks.

Deep learning can leverage neural networks to extract high-order features from unstructured data^10^, enabling an end-to-end working model from raw data input to result in output. It has a wide range of applications in fields such as natural language processing, medical image analysis, and financial data forecasting.

In response to the issues associated with traditional DDoS attack detection methods in SDN, we propose a two-stage attack detection and mitigation method based on deep learning by analyzing the organization form and traffic characteristics of DDoS attacks in SDN. In the attack detection phase, we first use the changes in statistical information from switch ports to make a preliminary judgment on the location of the attack source. Then, we conduct feature extraction based on the traffic data output from the suspicious switches, and further extract feature information in the “time, frequency, and spatial” domains of the input feature data using wavelet decomposition and convolutional neural network technology to classify the feature data with a classification function. In the mitigation phase, we utilize graph theory and dynamic deletion strategies to trace and isolate the attack source to mitigate the further adverse impact of the attack on the SDN.

### Contributions

The main contributions of our work are:

- A two-stage attack detection mechanism was designed, which achieves a preliminary detection of attack behaviors in the network by collecting statistical feature information from switches without adding extra blocks; further detection of traffic features from suspicious switches is conducted to achieve fine-grained detection of attack traffic.
- A multi-scale anomaly detection module was designed, utilizing wavelet transform and convolutional neural networks to extract multi-dimensional feature information from traffic data, and using a simple classifier to complete the identification and detection of anomalous traffic.
- Utilizing graph theory knowledge and restriction-based mitigation strategies, trace and isolate the attack source host, thereby preventing new attack traffic from entering the network, mitigating the impact of network attacks on SDN, and ensuring the normal operation of the network.

The rest of this paper is organized as follows. “Related work” section introduces the main organizational forms of DDoS attacks targeting SDN and provides a review of current research on DDoS attack detection and mitigation in SDN; “Deep learning-based attack detection and mitigation” section provides a detailed introduction to the attack detection and mitigation method based on deep learning; “Experimental results and analysis” section conducts detection experiments and analysis on the proposed method; Finally, “Conclusion” section concludes the paper.

## Related work

**Figure 1:**
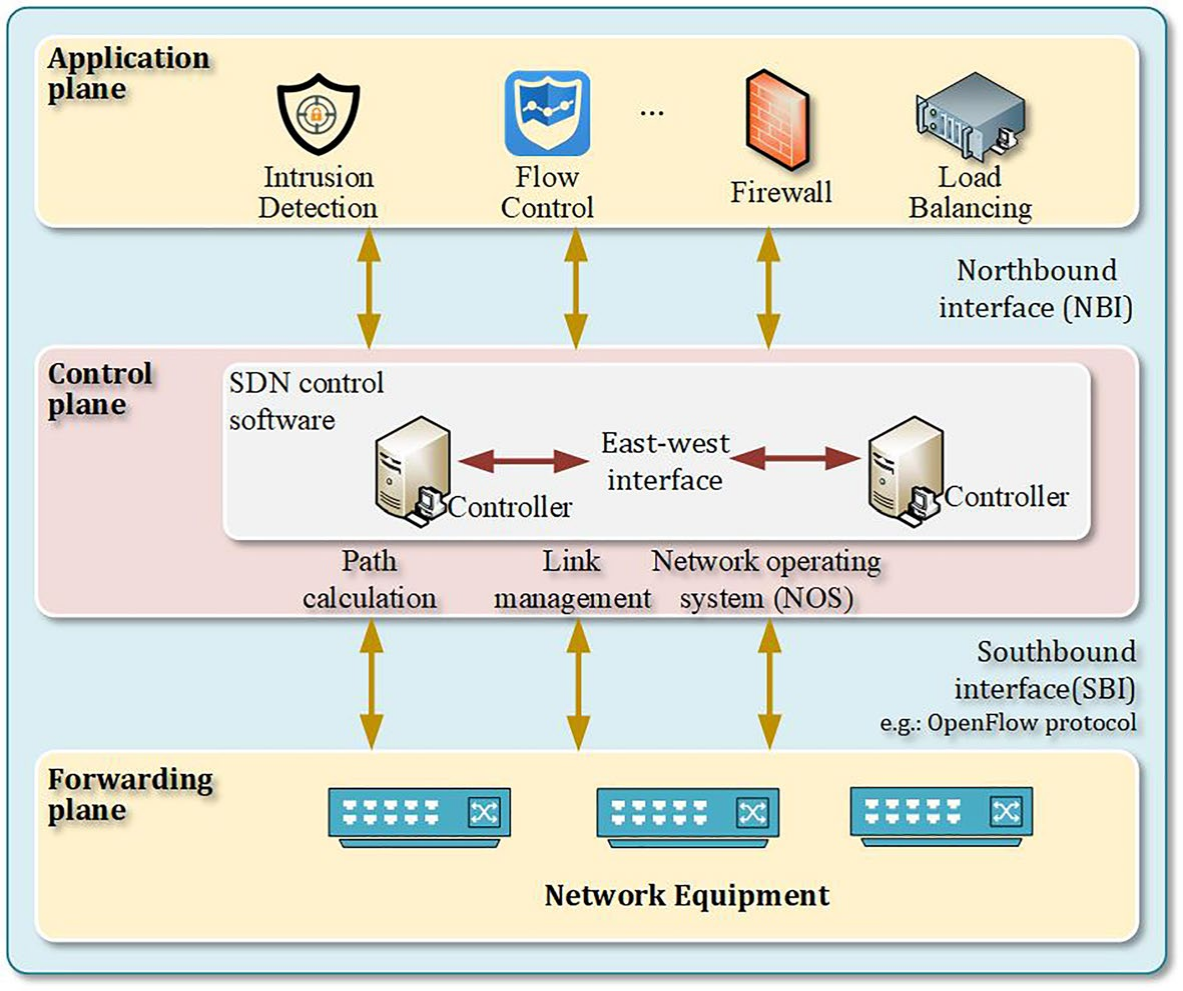
SDN architecture schematic diagram.

The SDN architecture consists of three main components: the application plane, the control plane, and the forwarding plane, as shown in Fig. 1. The application plane primarily serves users and typically includes network services and applications such as traffic control, load balancing, and intrusion detection. The control plane, which is composed of controllers, is responsible for establishing forwarding rules and managing the forwarding devices. It connects to the application plane via a northbound interface and responds to the application plane's requests. The forwarding plane, composed of network devices such as switches and routers, connects to the control plane via a southbound interface and executes the forwarding rules defined by the control plane. It also regularly reports network status information back to the control plane. An SDN network can have a single controller or multiple controllers, which are interconnected through an east-west interface. A single controller can manage multiple forwarding devices, and a single forwarding device can be controlled by multiple controllers. The open design of SDN offers broad application prospects, but it also faces the threat of emerging and ever-changing network attacks. Research teams from both domestic and international sources, in response to the characteristics of DDoS attacks on SDN, have proposed some targeted detection and mitigation methods. This section primarily combs and summarizes the common forms of DDoS attacks in current SDN environments, as well as some of the more popular methods for attack detection and mitigation. Open design of SDN offers broad application prospects, but it also faces the threat of emerging and ever-changing network attacks. Research teams from both domestic and international sources, in response to the characteristics of DDoS attacks on SDN, have proposed some targeted detection and mitigation methods. This section primarily combs and summarizes the common forms of DDoS attacks in current SDN environments, as well as some of the more popular methods for attack detection and mitigation.

### Organizational forms of DDoS attacks in SDN

The OpenFlow protocol is currently the most widely used southbound interface technology in the field of SDN. Typically, an OpenFlow switch always sends the relevant information of any new flow it receives to the controller for instructions on how to handle it. As a result, most DDoS attacks in SDN exploit vulnerabilities in the OpenFlow protocol^11^. Firstly, since the control plane is situated between the application plane and the forwarding plane, providing a programming interface to the upper layer and controlling hardware devices to the lower layer, if the control plane is compromised, the entire SDN can be affected. Therefore, the controller is the preferred target for DDoS attacks. Attackers often send a large number of new flows with random headers to the switch. Because these flows lack matching rules in the switch's flow table, the switch continuously queries the controller for a handling method. This causes the controller's query queue to grow continuously, resulting in the controller remaining constantly busy and unable to provide services to legitimate users^12^. Secondly, SDN switches are also a primary target for network attacks. According to the protocol, the controller generates a matching rule for each new flow request sent by the switch, and this rule is appended as a flow table entry to the flow tables of all switches that the packets from the source host to the destination host pass through, facilitating subsequent forwarding operations. However, due to the limited storage space of the switch, an excess of forwarding rules can cause the switch's flow table to overflow, preventing the switch from providing forwarding services for new legitimate flows^13^. Additionally, when a large number of packets flood the switch, exceeding its processing capacity, "packet loss" can occur, which affects the normal transmission of traffic data in the network^4^. Finally, because OpenFlow lacks the security protection mechanisms of the traditional network transport layer, the controller and the switch can establish a connection merely through an address. Therefore, attackers can also paralyze the SDN by modifying rules to reconfigure downstream switches and carry out more granular malicious attacks^14^.

### Detection of DDoS attacks in SDN

Current detection methods for DDoS attacks in SDN are largely adapted from those used in traditional networks, but they often perform unsatisfactorily when faced with the SDN environment. For instance, although statistical-based detection methods do not require prior knowledge and can perform detection, they necessitate appropriate distribution assumptions for traffic data beforehand, which does not adapt well to the dynamic network model of SDN. Information theory-based methods, while not requiring distribution assumptions, demand a large number of stable and reliable samples to ensure detection accuracy, which contradicts the random dynamic nature of SDN; Clustering-based detection methods are straightforward to implement but time-consuming, failing to meet the SDN's demand for timely detection. Therefore, against the backdrop of current big data, there is a growing interest in research on machine learning-based detection methods, such as Random Forests, Bayesian Networks, Support Vector Machines, and Multilayer Perceptrons.

Alduailij et al.^15^ utilized a combination of information gain and the Random Forest method to select the main features of traffic data, enhancing the accuracy of the model in detecting DoS attacks within an SDN environment in the cloud. Luo Zhiyong et al.^16^ proposed a Bayesian Attack Graph-based method for recognizing intrusion intentions in SDN. They first employed the PageRank algorithm to determine the criticality of devices, then combined attributes such as vulnerability value, attack cost, benefit, and preference to construct an attack intention table, using a risk assessment model to predict intrusion paths. Santos et al.^17^ used the Mininet program to set up an SDN environment, simulated DDoS attacks with the Scapy tool and IP lists, and compared the effectiveness of four machine learning algorithms—Support Vector Machine, Decision Tree, Random Forest, and Multilayer Perceptron—in detecting DDoS attacks, concluding that the Decision Tree-based detection method was the most effective. However, when facing large-scale network traffic, the detection capabilities of machine learning-based methods are not always satisfactory. Elsayed et al.^18^, through comparative analysis of several machine learning-based detection methods, found that the lack of labeled samples and weak feature correlation were the main reasons for the poor detection performance. They believe that deep learning, capable of reconstructing the unknown distribution of input data using multi-layer neural networks, has a good representational ability for large-scale network traffic. Therefore, an increasing number of scholars are beginning to focus on research into deep learning-based detection technologies.

Deep learning is a form of machine learning that supports neural network algorithms and is also a quintessential representation learning technique. It has a strong capacity for representing raw data and has been extensively applied in fields such as natural language processing, machine vision, and financial data analysis. There are also numerous practical applications in the realm of attack detection for SDN. ElSayed and colleagues^19^ have improved regularization methods for Convolutional Neural Networks (CNN), developing a novel SDN intrusion detection system that effectively mitigates the issue of overfitting that is common in deep learning models. Gadze and others^20^ have put forward an adversarial detection and defense approach for DDoS attacks within the SDN environment. This approach combines Generative Adversarial Networks, Deep Belief Networks, and Long Short Term Memory networks (LSTM) to effectively reduce the sensitivity of the detection model to adversarial attacks and to expedite the feature extraction process. Kachavimat et al.^21^ constructed a DDoS attack detection model that adapts to various deep learning architectures and conducted experiments on the InSDN^22^, the SDN-dataset, and DDoS attack data generated from the Mininet Ryu network. They concluded that the detection method based on Long Short-Term Memory (LSTM) outperforms Convolutional Neural Networks (CNN) and Multilayer Perceptrons in terms of overall effectiveness. Interestingly, in the same year, Lee et al.^23^, in their designed attack detection framework, compared the effectiveness of four deep learning detection models: Multilayer Perceptrons, CNN, LSTM, and Stacked Autoencoders. They believe that the detection effect of Multilayer Perceptrons is the best. However, current deep learning-based attack detection methods in SDN mostly inherit the detection ideas and methods from traditional networks. There is redundancy in the selection of feature data, which brings additional costs to the detection computation. This is because some features used in the detection, such as the number and size of packets, can be directly obtained by the controller from the forwarding layer. Moreover, most current detection methods are based on a single architecture and do not fully exploit and utilize the higher-order information of feature data, leading to suboptimal detection performance.

### Mitigation of DDoS attacks in SDN

After detecting a DDoS attack, how to eliminate or mitigate the impact of the attack on network service quality is another issue of concern for cybersecurity professionals. Overall, there are currently two main approaches to solving this issue: restricting the transmission capabilities of the attacking host and load balancing on network devices. Specifically, restricting the transmission capabilities of the attacking host does not mean completely discarding the data sent by the host, but rather assigning a higher forwarding priority to legitimate normal traffic and a lower forwarding priority to illegitimate traffic. This approach reduces the intensity and volume of DDoS attack traffic entering the SDN network, thereby ensuring that normal network services are not severely affected. However, this method cannot completely prevent attack traffic from entering the SDN network. Yungaicela et al.^24^ proposed an attack mitigation scheme based on deep reinforcement learning, which prioritizes data flows according to the controller's response time to users. This allows legitimate data flows to receive high-quality routing and forwarding, while malicious data flows are directed to special forwarding paths or are discarded outright. However, this method may inadvertently affect legitimate traffic with longer durations. Cao et al.^25^, on the other hand, combined the white list with the dropping strategy, discarding traffic that falls outside the white list directly. This approach reduces the load on the southbound interface and CPU overhead, but it may also inadvertently injure unknown normal traffic.

Additionally, load balancing on network devices involves dynamically adjusting the task distribution between controllers and switches, migrating network tasks from heavily loaded devices to lightly loaded ones to mitigate the impact of DDoS attacks on SDN service quality^26^. Filali et al.^27^ utilized game theory concepts, transforming the controller and switch allocation problem into a many-to-one matching game problem. They dynamically assign switches to controllers, ensuring that each controller meets a specified minimum quota, thus achieving a balance in network load. Although load balancing methods can alleviate the impact of DDoS attacks by equalizing the load on controllers, these methods cannot prevent switches from continuing to be subjected to DDoS attacks.

### Deep learning-based attack detection and mitigation

#### System overview

**Figure 2:**
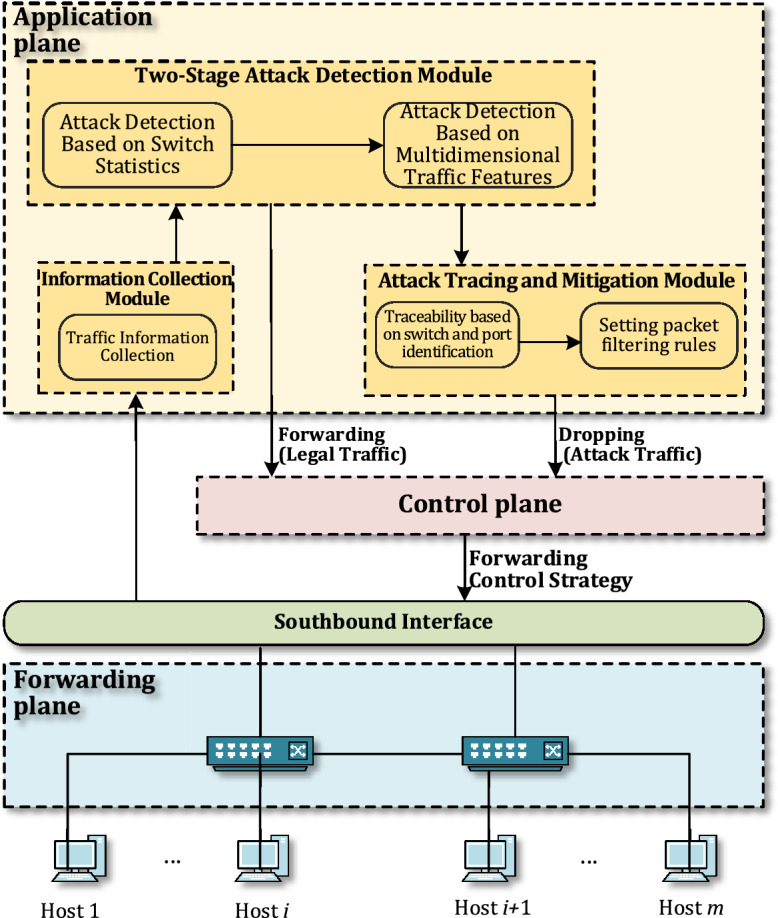
Overall architecture of the attack detection and mitigation system.

The system we designed for DDoS attack detection and mitigation in SDN based on deep learning belongs to the application layer services and can be deployed on devices within the application plane or on the server where the SDN controller resides. The system consists of a traffic information collection module, a two-stage attack detection module, and an attack source tracing and mitigation module, as shown in Fig. 2.

The system monitors the traffic data in the SDN in real time and performs a preliminary detection of network attacks based on the statistical information from the switch ports. It then utilizes wavelet decomposition and convolutional neural network technology for depth analysis of the traffic data from suspicious switches, enabling fine-grained detection of attack traffic. Finally, by employing graph theory and dynamic deletion strategies, the system tracks and restricts the source of the attack, preventing the attack traffic from entering the network, and thereby ensuring the normal operation of the SDN.

The purpose of the information collection module is to periodically collect relevant information on the traffic data passing through the switch ports, transform it into the required format, and then send it to the two-stage detection module. The first stage of detection only requires extracting some rough count information of the data packets and flows passing through the switch. The second stage of detection, however, requires the use of specialized traffic analysis tools to extract traffic information that has been aggregated based on the five-tuple characteristics (source IP, source port number, destination IP, destination port number, protocol) of the flows.

The two-stage attack detection consists of attack detection based on switch statistics and attack detection based on multi-dimensional traffic features. In the first stage, which is the attack detection based on the statistical information of switch port traffic, the primary task is to perform a preliminary detection of DDoS attacks within the network segment controlled by the switch. We know that when a DDoS attack is launched, the switch connected to the attacking host will receive a large number of forwarding requests for new flows. Since there are no matching flow entries in the switch's flow table, the switch will send a large number of PacketIn messages to the controller to obtain disposition methods for these new flows. Therefore, the ratio of the number of flows received by the switch to the number of forwarded packet messages within a unit of time will suddenly decrease compared to normal conditions, and the ratio of the number of normal forwarding flows to the number of received flows will also decrease. Additionally, under normal network conditions, the number of incoming and outgoing data packets is relatively balanced, with not much difference between them. However, when a switch is under a DDoS attack, a large volume of packets arrives at the switch in a short period and cannot be forwarded promptly, leading to temporary storage in the buffer. If the buffer space is exhausted, a "packet loss" phenomenon occurs^28^ at which point the network exhibits a significant discrepancy between the number of incoming and outgoing packets. When several traffic characteristic indexes exceed the critical value, it can be judged that there is an attack behavior in SDN, and at the same time, the rough location of the attack source can be completed.

The second stage is an attack detection based on multi-dimensional traffic characteristics, which is initiated when the first stage detects certain switches exhibiting attack behaviors. Initially, a traffic collection program captures all the traffic data passing through the suspicious switch. Then, data analysis tools are used to extract the characteristics of the traffic. Subsequently, wavelet transform is utilized to extract the time–frequency characteristics of the traffic data at different scales, and a Convolutional Neural Network (CNN) is employed to extract the spatial characteristics of the data. Finally, a classifier is used to categorize these rich feature data, thereby achieving the detection of attack traffic.

The attack mitigation module is started after detecting the attack flow in the second stage. It locates the attack source according to the state information of the attack source host provided by the attack detection module, formulates the data packet filtering rules including IP address, port number, effective time, execution action, etc., and sends them to all switches under its control through the controller. The switch updates its flow table according to the new rules issued by the controller, deletes the attack flow entries in the flow table, and introduces all new flows without matching rules sent by the attack host to the default port for discarding within the set effective time, to achieve the goal of preventing the spread of network attacks. At the end of the set time, if no new restriction rule is received, the host is restored to send a new stream. In this process, the attack detection module continuously monitors the network state and transmits the detected new attack information to the attack mitigation module in time, and the attack mitigation module continuously generates new packet filtering rules and sends them to the switch; The switch updates its flow table according to the new rules, and handles the traffic data in the network according to the new flow table, thus realizing the uninterrupted detection and protection of SDN.

### Two-stage attack detection

Two-stage attack detection is the basis of attack mitigation and the key to maintaining the safe operation of SDN. As the core content of this paper, before describing two-stage attack detection in detail, the symbols used in this section are explained, as listed in Table 1.

**Table 1:** List of common symbols.

Symbol	Meaning
$NFI$	Number of network traffic flowing into the switch
$NFO$	Number of network traffic flowing out of the switch
$NPi$	Number of PacketIn packets forwarded from the switch to the controller
$NPI$	number of packets into the switch
$NPO$	Number of packets out of the switch
$RPi$	The ratio of the number of network traffic flowing into the switch to the number of PacketIn forwarded from the switch to the controller. (inflow-forwarding ratio)
$RFI$	The ratio of the number of network traffic out of the switch to the number of normal network flows flowing into the switch. (normal forwarding ratio)
$\Delta NP$	Difference in the number of input and output switch packets

1. Attack detection based on switch statistics

We periodically collect statistics at the switch's ports on the number of network flows and data packets entering and exiting the switch, as well as the number of PacketIn messages forwarded by the switch to the controller. Under normal circumstances, flows with corresponding matching rules in the switch's flow table can be processed normally, while flows without matching rules need to inquire with the controller for handling methods. However, the number of such flows is generally not high. Therefore, there are $NFI$ >  > $NPi$ in SDN switches, and the ratio between them is a relatively large value. However, when a DDoS attack is launched, a large number of new flows will arrive at the switch to request forwarding operation in a short time. Because these flows are all new, and there is no corresponding matching rule in the switch flow table, each incoming new flow will generate a PacketIn message sent to the controller, so the switch will send a large number of PacketIn messages to the controller to ask how to deal with them. At this time, the ratio of the number of network flows flowing into the switch to the number of PacketIn forwarded from the switch to the controller will be much smaller than normal, and $RPi$ can be expressed as follows:

1 $$RPi = \frac{NFI}{{NPi}}$$

In addition, even if there is a DDoS attack in the network, those normal flows with matching rules in the switch can still be forwarded correctly before the switch is completely blocked, and their proportion in all network flows flowing into the switch is:

2 $$RFI = \frac{NFO}{{NFI}}$$

After the attack is launched, the $RFI$ metric of the switch will experience a very noticeable decline.

Furthermore, when a DDoS attack occurs, a large number of data packets arrive at the switch in a short time and cannot be forwarded in time, so they can only be temporarily stored in the cache of the switch; However, the buffer space of the switch is limited, and once it is filled, a large number of packets will be lost, which will lead to the difference between the number of packets flowing into and out of the switch

3 $$\Delta NP = \left| {NPI - NPO} \right|$$

Compared with the normal network situation, it has a significant increase.

To sum up, when both $RPi$ and $RFI$ of a switch are reduced to a certain threshold and $\Delta NP$ is increased to a certain extent, it can be preliminarily determined that there is DDoS attack in the network; And the switch is the transmission node of the attack flow in SDN, and the attack source must be on the link to which the switch is connected.

(2) Attack detection based on multi-dimensional traffic characteristics

**Figure 3:**
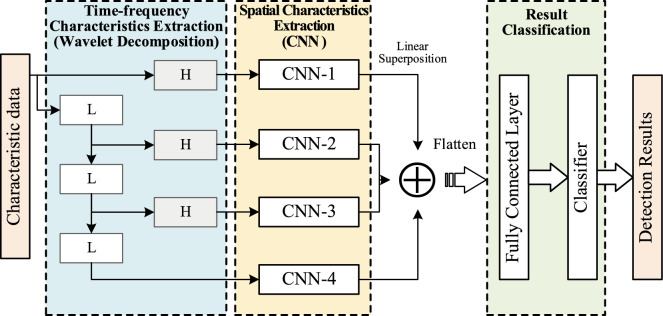
Attack detection process based on multi-dimensional traffic characteristics.

The detection in this phase relies on the Multi-Dimensional Deep Convolution Classifier (MDDCC) to complete, which is initiated when an anomaly is detected in a certain switch during the first phase. Initially, the traffic capture program Wireshark is used to intercept all the traffic data passing through the suspicious switch. Subsequently, the CIC-FlowMeter analysis tool is utilized to extract the characteristics of the traffic. After the data is preprocessed, it is then sent to the MDDCC to achieve precise detection of the abnormal traffic. MDDCC is a traffic classification model that combines wavelet transform technology with deep learning. It is capable of using wavelet transform to extract the time–frequency characteristics of traffic data and using CNN to extract the spatial characteristics of the data. The model conducts a comprehensive analysis of the traffic data from three dimensions: "time, frequency, and space". Finally, the classification of the data type is completed by the SoftMax classification function, and its structure is shown in Fig. 3. Due to the adoption of parameter sharing, local perception, and pooling operations, the training parameters and training time of CNN are significantly reduced compared to traditional multi-layer perceptrons.

We know that the temporal correlations hidden within sequential data are closely related to frequency. Correlations of information on larger time scales, such as the long-term trends inherent in the data, are typically found in the low-frequency range. In contrast, correlations of information on smaller time scales, such as the characteristic information resulting from short-term disturbances or sudden random events, are usually located in the high-frequency range. To thoroughly explore the correlations within traffic sequences, we apply wavelet decomposition to the input sequence ${\text{x}} = \left\{ {{x_1}{,}{x_2}{,} \ldots ,{x_k}} \right\}$, which allows us to obtain its low-frequency component ${{\text{x}}^l}(i)$ at the ith level and its high-frequency component ${{\text{x}}^h}(i)$. They are respectively as follows:

4 $${{\text{x}}^l}{(}i{)} = \left\{ {x_1^l{(}i{), }x_2^l{(}i{)}, \, \ldots {, }x_k^l{(}i{)}} \right\}$$

5 $${{\text{x}}^h}{(}i{)} = \left\{ {x_1^h{(}i{), }x_2^h{(}i{)}, \ldots \, x_k^h{(}i{)}} \right\}$$

Because we only use the decomposition sequence of the original sequence, we don't need wavelet reconstruction, so we don't need to adopt downsampling when we decompose again. After decomposition for $n$ times, the $n + 1$ subsequence with the same dimension as the original sequence can be finally obtained, and the sequence set can be expressed as follows:

6 $$\chi (n) = \left\{ {{x^h}(1), \, {x^h}(2), \, \ldots , \, {x^h}(n), \, {x^l}(n)} \right\}$$

Each subsequence is converted into a two-dimensional graphic format and input into n + 1 independent CNN for spatial feature extraction, and each subsequence is subjected to a series of convolution operations to obtain the results.

7 $${z^i} = g({x^i} \otimes \omega + b) \, i \in [0,n]$$

Here, ${x^i}$ represents the subsequence obtained after the ith level of wavelet decomposition, which is also the input to the ith CNN. ${z^i}$ is the output subsequence after the CNN transformation. $\omega$ and $b$ represent the weights and biases, respectively, $g( \cdot )$ is the nonlinear activation function, and $\otimes$ denotes the convolution operation. We use the mean squared error as the loss function. Additionally, since traditional L1 and L2 regularization methods only focus on individual feature weight values without considering the intrinsic connections between feature values, we employ a regularization method based on the standard deviation constraint operator to prevent overfitting issues.

8 $$\sigma (\omega ) = \sqrt {\frac{1}{nk}\left\{ {\sum\limits_{i = 1}^{nk} {\omega_i^2} - \frac{1}{nk}{{\left( {\sum\limits_{i = 1}^{nk} {\omega_i} } \right)}^2}} \right\}}$$

$k$ represents the number of rows in the weight matrix, $i$ denotes the *i*th row of the weight matrix, and $n$ is the number of columns in the weight matrix, which is the size of the weight vector. The value of the weight matrix is controlled by $\lambda$, so the loss function *L* can be expressed as follows:

9 $$L = {\min_\omega }\{ f(X,y:\omega ) + \lambda \sigma (\omega )\}$$

Therefore, we minimize the loss function related to $\omega$ by standard deviation.

Finally, the output subsequences are linearly superimposed and expanded, then inputted into a fully connected layer for computation. Subsequently, the SoftMax classification function is used to complete the classification of the input sample data.

10 $$y^{\prime} = Classifie{r_{softmax}}(Compose_{i = 0}^n({z^i}))$$

### Attack traceability mitigation module

Before mitigating the attack effect, the attack path discovery strategy based on graph theory and switch and its port identification is used to locate the entrance switch of the attack host accessing the SDN network according to the transmission path of the attack stream. The path of network traffic in SDN can be expressed as follows:

11 $${E_{i,j}} = \sum {({s_i},{p_i})} \to ({s_j},{p_j})$$

Among them, ${E_{i,j}}$ represents the transmission path of the network flow, ${s_i}, \, {s_j}$ are the node switches on the transmission path of the network flow, and ${p_i}, \, {p_j}$ are the port numbers of the switches, respectively. When the network attack traffic passes through the two switches ${s_i}, \, {s_j}$ the edge connecting ${s_i}$ and ${s_j}$ is considered to be the attack path, and ${p_i}, \, {p_j}$ are the interfaces through which the attack traffic enters and exits. By combining this with the controller's grasp of the SDN's global topology, the attack can ultimately be located at the edge switch and the access port through link tracing. It can be seen that this traceability method does not use information such as IP address and Mac address inside the packet, so even if the attacker uses forged address information, it can still be accurately traced to the interface port position.

After tracing the switch and port where the attacking host accesses the SDN, a restriction policy is implemented for the host connected to the switch port, that is, within a certain time, any packet with no matching rules sent from the attacking port is discarded while prohibiting this switch from sending the PacketIn to the controller. By this method, the new flow request of the attacking source can be isolated, thus preventing the new attack flow from entering the network. The implementation process is shown in Algorithm 1.

**Figure:**
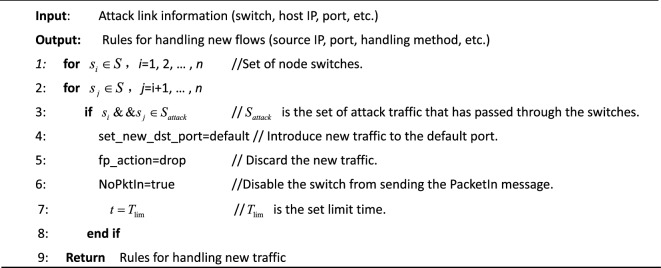
Algorithm 1. Algorithm of DDoS attack mitigation

After the forbidden time exceeds the set time, the forwarding function of the switch is restored. Additionally, although the attack traffic is intercepted, the previous flow table entries are still stored in the switch flow table, which will affect the normal forwarding process of the network and always consume the resources of the controller and the switch. Therefore, for the flow detected as an attack, the controller uses its host tracking function to obtain the relevant information of the attacking host, such as MAC address, IP address, TUP or UDP port, switch port, etc. At regular intervals, a dynamic deletion policy is generated and sent to the switch, and the switch deletes the relevant entries in the flow table entry. This method can effectively restrict the attack flow on the attack link without affecting the forwarding of other normal flows.

## Experimental results and analysis

### Experimental settings

**Figure 4:**
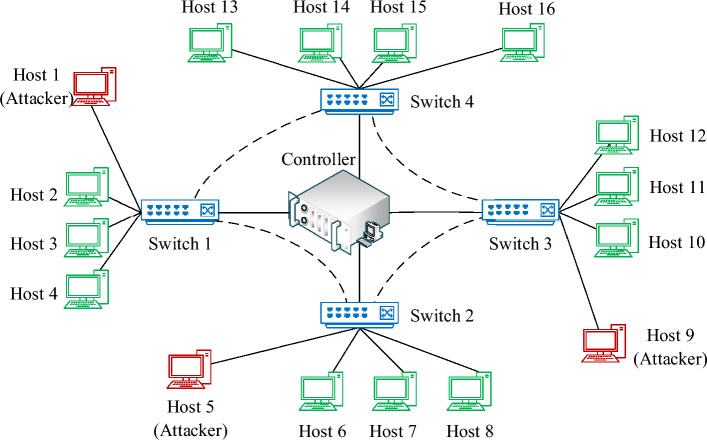
SDN network topology diagram.

The hardware configuration of the experimental platform is Intel Core i9-12900F, 128 GB RAM, and NVIDIA RTX3090. The detection system is written in Python language, adopts Pytorch1.8 deep learning framework and runs on the Ubuntu 16.04 LTS operating system. In addition, using the Mininet simulator and POX controller to build an SDN environment, the network topology is shown in Fig. 4.

Mininet simulates 4 switches and 4 hosts connected to each switch, which are connected to a POX controller to form a star-shaped network structure. The network delay is set to 2 ms, and the IP address information for the controller server and each host is shown in Table 2.

**Table 2:** Controller and host address information.

Name	IP address	Role
Controller	192.168.100.1	Traffic control
Host 1	192.168.10.1	Attacker
Host 2–4	192.168.10.2-4	Normal user
Host 5	192.168.20.1	Attacker
Host 6–8	192.168.20.2-4	Normal user
Host 9	192.168.30.1	Attacker
Host 10–12	192.168.30.2-4	Normal user
Host 13–16	192.168.40.1-4	Normal user

Host 1, Host 5, and Host 9 are hosts that launch DDoS attacks, and the attack program is Hping3 network tools. When attacking, several attacking hosts send a large number of TCP-SYN packets to the network with randomly generated target IP addresses to simulate DDoS attacks. In addition, other hosts, as normal users, run the Distributed Internet Traffic Generator (D-ITG) program to generate background traffic. The data transmission rate includes constant distribution, uniform distribution, exponential distribution, Poisson distribution, and gamma distribution, and the size of the data packets they generate all obey Poisson distribution.

### Data details

1. Data characteristic selection

Processing raw data is an advantage of deep learning; however, this advantage requires strong high-performance computing power for support, and it will also consume more computational time. To reduce computational pressure and shorten detection time, based on the research results of Krishnan et al.^29^, we use Wireshark to capture raw traffic data in .pcap format, and then select 48 features from over 80 features obtained from the CIC-FlowMeter analysis tool as the experimental data for model training and detection.

2. Data preprocessing

Usually, there may be issues such as missing feature values, format errors, and significant differences in units of measure in the traffic data, all of which can affect the effectiveness of detection. Therefore, before inputting into the attack detection model, it is necessary to perform certain preprocessing operations on the detection data, which mainly include data cleaning, feature value encoding, and data normalization. Data cleaning primarily eliminates data samples in the dataset that have missing feature values. If the sample missing rate is very high (greater than 80%) and of low importance, it is directly deleted; if the missing rate is not significant and the data is relatively important, the mean imputation method is used to repair the data, ensuring the completeness of the data samples. Feature value encoding mainly involves encoding non-numeric feature values into recognizable numerical values for the computer, with text adopting the one-hot encoding method. Numerical standardization is mainly conducted to eliminate the adverse impact of excessively large dimensional differences of data on the detection results, which involves scaling the data by a certain proportion. In this study, the Min–Max standardization method is employed. After standardization, all characteristic values are mapped within the interval [0,1], with the maximum characteristic value being 1 and the minimum feature value being 0.

### Model parameters setting

The MDDCC proposed in the “Two-stage attack detection” section employs a design that integrates wavelet transform with convolutional neural networks (CNN). The wavelet basis function utilizes the Daubechies wavelet (DB), with a decomposition level of 3. The CNN consists of 3 convolutional layers, using $3 \times 3$ convolutional kernels, followed by a $2 \times 2$ max pooling layer after each convolutional layer. Dropout is utilized to prevent overfitting. The loss function is the mean-square error (MSE), and the model parameters are updated using the mini-batch gradient descent method and the backpropagation (BP) algorithm. The specific hyperparameter settings are as shown in Table 3.

**Table 3:** MDDCC superparameter.

Parameter name	Parameter value
Wavelet basis function	DB4
Wavelet decomposition level	3
CNN-convolution kernel	$3 \times 3$
CNN-activation function	Relu
CNN-convolution layer output	32, 64, 32
CNN-dropout	0.2, 0.3, 0.2
Max-pooling	$2 \times 2$
Echo	100
Normalization	L2
Learning rate	0.01

### Evaluation metrics

To evaluate the performance of the detection method against network attacks, we use five detection metrics as references to assess the performance of the detection method: Accuracy, Precision, Recall, F1 score, and False Positive Rate. Their calculation methods are as follows.

12 $$Accuracy = \frac{TP + TN}{{TP + FP + FN + TN}}$$

13 $$Precision = \frac{TP}{{TP + FP}}$$

14 $$Recall = \frac{TP}{{TP + FN}}$$

15 $$F1 = \frac{2 \times Precision \times Recall}{{Precision + Recall}}$$

16 $$FPR = \frac{FP}{{FP + TN}}$$

Among them, the relationships between TP (True Positives), FN (False Negatives), FP (False Positives), and TN (True Negatives) can be represented by the confusion matrix in Table 4.

**Table 4:** Matrix of the relationship between true value and predicted value.

	Positive detection	Negative detection
True positive	TP	FN
True negative	FP	TN

### Experimental results and analysis

The experiment is divided into two phases: The first phase mainly verifies the performance of the attack detection method based on switch statistics, while the second phase primarily evaluates the detection performance of MDDCC on attack traffic.

#### Attack detection based on switch statistical information

**Figure 5:**
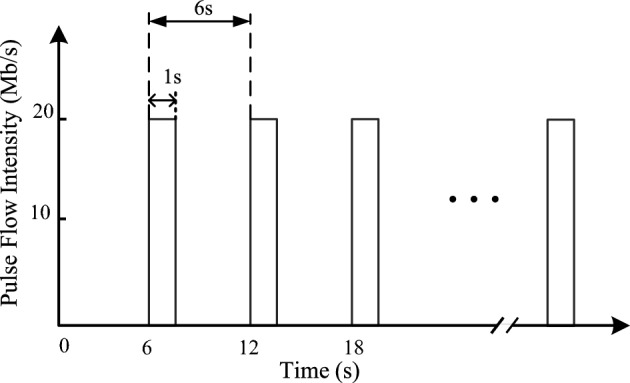
Schematic diagram of pulse DDoS attack.

In this attack detection experiment, host 1 is set as the attack host, and Hping3 is used to continuously send SYN pulses with the intensity of 20 Mb/s to switch 1, each pulse lasts for 1 s, and then it is silent for 5 s, that is, the period of the attack pulse is 6 s. Figure 5 is a schematic diagram of a pulsed DDoS attack.

Other hosts simulate normal users, each sending normal data packets according to the distribution pattern preset in the configuration strategy. The traffic information collection module continuously collects information from each switch port with a period of 1 s and transforms it into the data pattern required for detection, inputting it into the anomaly detection module. The attack experiment lasts for 6 h, during which each switch collects 21,600 samples. Specifically, switch 1 has 18,000 normal samples and 3,600 attack samples; Switch 2, Switch 3, and Switch 4 all have normal samples only. Calculate the $RFI$, $RPi$, and $\Delta NP$ feature values for each switch, respectively. Since the network latency is minimal and can be disregarded, it can be determined that there is an attack behavior in the network when all feature values exceed their thresholds. Here, the threshold refers to the mean and standard deviation of the features $RFI$, $RPi$, and $\Delta NP$ calculated after sampling 10,000 sets when only normal traffic exists in the network. Then, following the "three-sigma (3σ)" rule, the thresholds for $RFI$ and $RPi$ are set to the mean minus three times the standard deviation, while the threshold for $\Delta NP$ is set to the mean plus three times the standard deviation.

**Figure 6:**
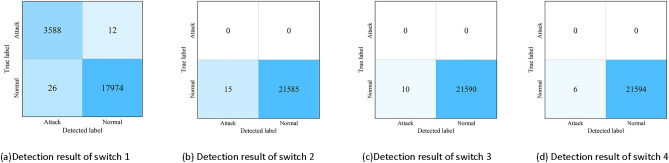
Results of abnormal detection for each switch.

Figure 6 illustrates the attack detection situation for 4 switches. It can be observed that Switch 1 has a large number of abnormal samples, which allows us to determine that this switch is abnormal.

The detection method in this phase achieved an accuracy of 99.82%, a precision rate of 99.28%, a recall rate of 99.67%, an F1 score of 99.47%, and an FPR of only 0.14% for detecting abnormal samples in the abnormal switch. It can be said that the attack detection method based on switch statistics can accurately pinpoint the switch connected to the host initiating a DDoS attack in SDN. Additionally, the reason why normal samples are judged as abnormal for other switches during detection is that although the traffic data sent by normal hosts follows a certain distribution, there may still be a sudden change in communication traffic at a certain moment, leading the detection system to mistakenly believe that there is a network attack behavior in the network where the switch is located.

In terms of detection time, Table 5 counts the time consumption of detecting each attack sample, and it can be found that the detection time is mostly within 100 ms, that is to say, when an attacker launches a DDoS attack, the detection system can find the abnormal behavior of the network and locate the location of the problem switch within 0.1 s, which can provide support for the real-time security protection research of SDN in the future.

**Table 5:** Detection time of attack samples.

Detection time consumed (ms)	Occurrence frequency
< 80	23
80–84	159
84–88	692
88–92	468
92–96	945
96–100	641
100–104	421
104–108	135
108–112	55
> 112	49

The detection method based on port statistics can quickly locate the switches through which the attack traffic passes. However, if protective measures are formulated solely based on such rough detection results, it could inadvertently harm other normal hosts connected to the switch and, in severe cases, may lead to partial network paralysis. Therefore, a more refined detection approach is necessary to provide accurate information about the source of the attack, which is essential for accurately and efficiently protecting the SDN network.

#### Attack detection based on multi-dimensional traffic characteristics

In this phase of the experiment, Host 1, Host 5, and Host 9 are set as attackers, running the Hping3 attack program to launch intermittent DDoS attacks on the SDN; other hosts continue to send normal TCP or UDP packets to the network according to the predetermined distribution pattern. Using the WireShark (v4.2) packet capturing tool, raw traffic data in .pcap format is obtained from the established SDN network experimental platform, and using the CIC-FlowMeter (v4.0) traffic analysis tool, the traffic data is aggregated and converted into .csv format feature data based on the five-tuple information of the flows. The converted dataset contains a total of 77,328 traffic records, with 36,642 records for normal flows and 40,686 records for attack flows. From each record's over 80 features, a subset of 48 features is extracted to form the detection dataset. The training set and the test set are formed by randomly sampling from the normal flow samples and attack flow samples in a 7:3 ratio, respectively.

- Detection performance of MDDCC

Using the training set, we conduct supervised training on the MDDCC, stopping when the loss function no longer decreases significantly due to training, and then fixing the model parameters. To eliminate random errors during detection calculations, we use the trained model to perform 5 independent detections on the test set data, with the test set samples being randomly shuffled before each detection. We calculate the five metrics for each detection and take the average values and deviations of the detection metrics as the final results of the MDDCC's detection of network attacks in SDN, as shown in Table 5.

Using the training set, we conduct supervised training on the MDDCC, stopping when the loss function no longer decreases significantly due to training, and then fix the model parameters. To eliminate random errors during detection calculations, we use the trained model to perform 5 independent detections on the test set data, with the test set samples being randomly shuffled before each detection. We calculate the five metrics for each detection and take the average values and deviations of the detection metrics as the final results of the MDDCC's detection of network attacks in SDN, as shown in Table 6.

**Table 6:** MDDCC's detection performance on the simulation dataset.

Detection serial number	Accuracy	Precision	Recall	F1	FPR
1st	0.9962	0.9983	0.9969	0.9976	0.0069
2nd	0.9963	0.9982	0.9972	0.9977	0.0073
3rd	0.9967	0.9985	0.9973	0.9979	0.0058
4th	0.9964	0.9983	0.9972	0.9978	0.0068
5th	0.9967	0.9985	0.9975	0.9979	0.0061
Mean ± standard deviation	0.9965 ± 0.0002	0.9984 ± 0.0013	0.9972 ± 0.0002	0.9978 ± 0.0001	0.0066 ± 0.0006

As can be seen from the table, the final detection accuracy of MDDCC is 99.65%, the Accuracy rate is 99.84%, the Recall rate of attack samples is 99.72%, and the F1 value is 99.78%. These indicators are above 99% in each test, and the deviation of each test result is very small, which shows that our MDDCC detection model based on multi-dimensional traffic characteristics can accurately and stably distinguish normal traffic and attack traffic in the SDN environment. In addition, the average false positive rate is only 0.66%, which is acceptable from the demand of current network system security protection tasks.

(b) Detection performance of MDDCC under different decomposition levels

**Figure 7:**
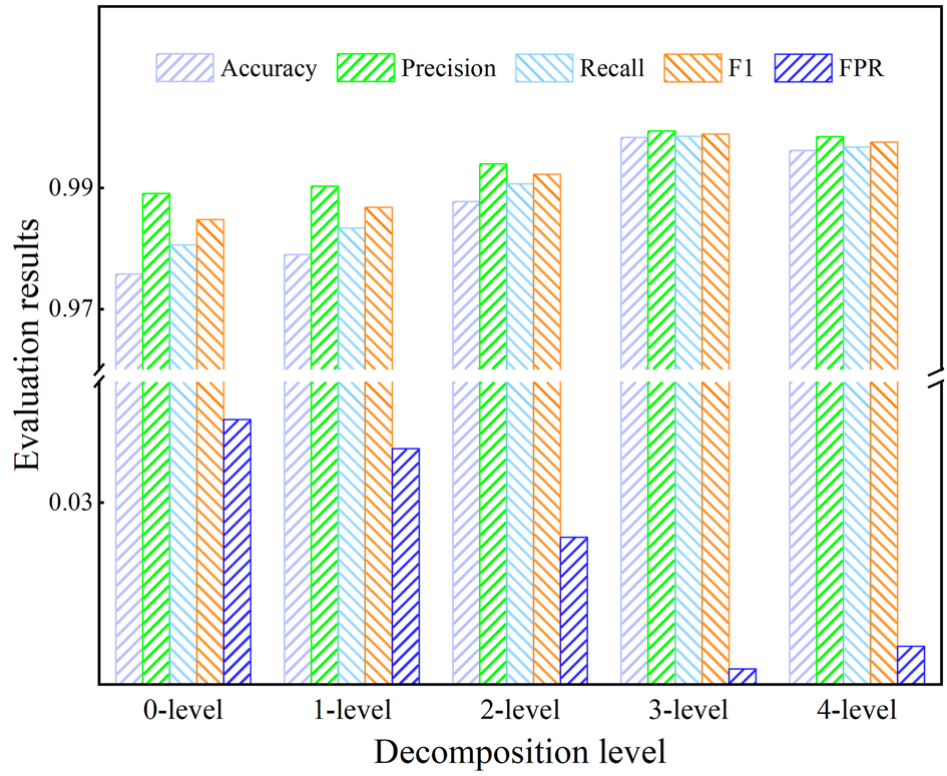
Detection performance of MDDCC under different decomposition levels.

In the previous experiment, MDDCC adopted a detection model with three-level wavelet decomposition. To explore the impact of wavelet decomposition on detection performance, the detection performance of MDDCC under different decomposition levels such as 0-level, 1-level, 2-level, 3-level, and 4-level wavelet decomposition was compared. The specific results are shown in Fig. 7.

It can be observed that as the decomposition level increases, the detection performance of MDDCC gradually improves. For instance, the precision metric of MDDCC at decomposition levels of 0, 1, 2, and 3, is 97.58%, 97.88%, 98.68%, and 99.84% respectively, showing a progressive increase; the Accuracy, Recall, and F1 score also follow this trend, and the FPR gradually decreases. This is because the higher the levels of wavelet decomposition, the richer the information that the feature sequence can provide. MDDCC can then discover more subtle differences between samples from features of different granularity, which helps to enhance the model's ability to identify attack samples. However, when the decomposition level is four, the precision metric of MDDCC is 99.62%, which is slightly lower than when the decomposition level is three. This is because the sequence data becomes overly decomposed, resulting in information redundancy. The ineffective features in the data, once amplified after being extracted by the deep neural network, reduce the performance of the classifier. It is evident that persistently increasing the level of wavelet decomposition does not provide additional effective feature information, and the improvement in model detection performance is limited, which is mainly determined by the amount of information contained within the original sample itself. Since the model achieves the best detection effect at a decomposition level of three, a three-level decomposition model is used for all following experiments.

(c) Detection performance in other data sets

To objectively assess the detection performance and generalization capability of MDDCC, comparative experiments were conducted using the public SDN dataset InSDN^29^. The InSDN dataset features normal and abnormal samples stored in separate files, and there is an imbalance in the categories of samples. Therefore, 70% of each category of samples was selected to form the training set, with the remaining 30% designated as the test set. Additionally, due to the scarcity of U2R samples (only 17 in total), which makes them unsuitable for participation in training and testing, they were excluded. The distribution of the samples in the re-divided InSDN dataset is illustrated in Table 7.

**Table 7:** Sample distribution of InSDN data set after division.

Sample type	Training set	Test set
DDoS	85,359	36,583
DoS	37,531	16,085
Probe	68,690	29,439
Brute-force-attack (BFA)	984	422
Web-attack	134	58
BotNet	115	49
Normal	47,897	20,527
Total	240,710	103,163

**Figure 8:**
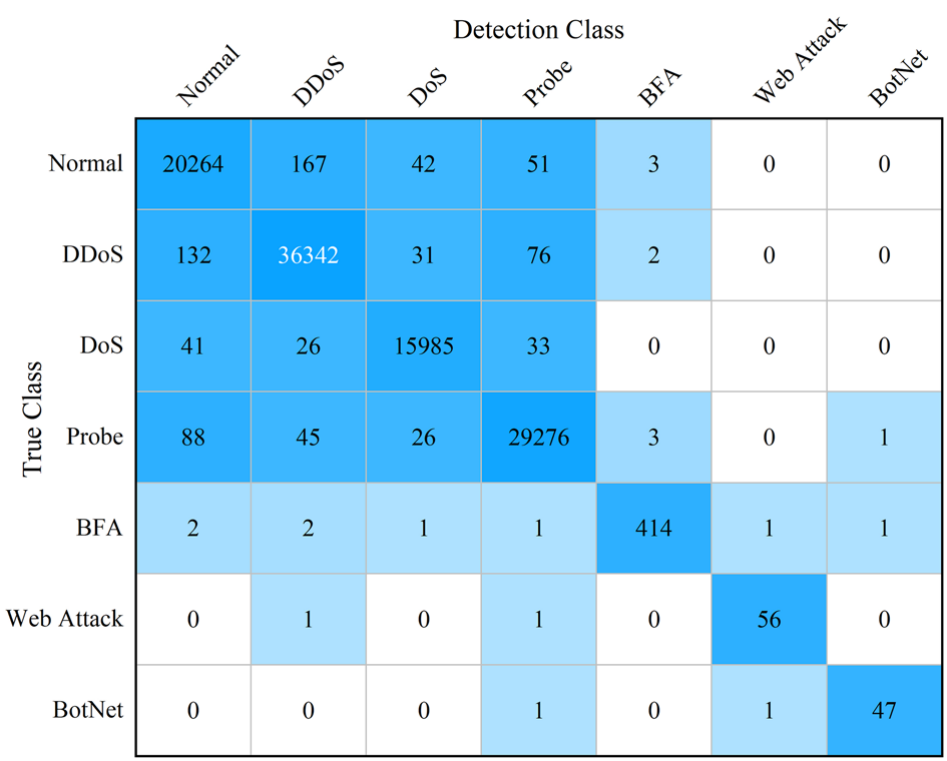
Classification performance of MDDCC on the InSDN dataset.

We utilized the divided training sets and testing sets, forming a subset of feature data using the 48 features selected as described in the “Data details” section for the model's training and testing. Due to the class imbalance of the samples, to prevent the model from developing a "preference" during training, a tenfold cross-validation method was employed for training MDDCC. This involved randomly dividing the training data into 10 groups, using 9 groups as the training data and 1 group as the validation data for each iteration. After completing 10 cycles of training, the model was fully trained using the complete training set data to achieve its final state. Finally, the preprocessed InSDN test set data was input into the well-trained MDDCC model for 5 complete tests, and the classification results as shown in Fig. 8 can be obtained.

From the figure, it can be observed that the MDDCC model exhibits varying detection capabilities for different types of attack samples. For instance, the recall rate is highest for DoS attack samples at 99.38%, while it is lowest for BotNet at 95.92%. When calculating from the perspective of the binary classification task of distinguishing between attack and normal samples, the MDDCC model achieves an average accuracy of 99.23%, precision of 99.68%, recall of 99.36%, F1 score of 99.52%, and a false positive rate of 1.28% on the InSDN test set. Overall, the MDDCC model's performance on the InSDN dataset is still quite impressive.

To further verify the generalization capability of MDDCC, we conducted experiments on two commonly used traffic datasets: CIC-IDS2017 and CIC-DDoS2019. These datasets, published by the Canadian Institute for Cybersecurity, simulate real-world network traffic by constructing 25 abstract user behaviors using protocols such as HTTP, HTTPS, FTP, SSH, email, etc. The attack traffic is generated by various cyber attack programs. Specifically, the abnormal traffic in CIC-IDS2017 is produced by seven types of attack behaviors: DoS, DDoS, Web Attack, Botnet, Brute Force, Heartbleed, and internal network penetration. The attack traffic in CIC-DDoS2019, on the other hand, is generated by reflection attacks targeting TCP (MSSQL, SSDP) and UDP (CharGen, NTP, TFTP) protocols, as well as SYN and UDP flood attacks that exploit vulnerabilities in these protocols. Both datasets provide. pcap format raw files as well as flow files containing more than 80 features generated by the FlowMeter traffic analysis tool. The experimental data was prepared following the method described in the “Data details” section, selecting 48 features to form the training and testing sets. During the training phase, the models were fully trained with their respective training sets until reaching a steady state. Finally, the models were tested for attack detection using their respective test sets for 5 trials. Table 8 presents the detection results of MDDCC on the CIC-IDS2017 dataset.

**Table 8:** Detection performance of MDDCC on the CIC-IDS2017 dataset.

Detection serial number	Accuracy	Precision	Recall	F1	FPR
1st	0.9958	0.9987	0.9960	0.9974	0.0052
2nd	0.9964	0.9988	0.9967	0.9978	0.0049
3rd	0.9961	0.9982	0.9969	0.9976	0.0073
4th	0.9957	0.9985	0.9966	0.9976	0.0061
5th	0.9956	0.9983	0.9962	0.9972	0.0068
Mean ± standard deviation	0.9959 ± 0.0003	0.9985 ± 0.0003	0.9965 ± 0.0003	0.9975 ± 0.0003	0.0060 ± 0.0010

Table 9 displays the detection results of MDDCC on the CIC-DDoS2019 dataset.

**Table 9:** Detection performance of MDDCC on the CIC-DDoS2019 dataset.

Detection serial number	Accuracy	Precision	Recall	F1	FPR
1st	0.9969	0.9819	0.9855	0.9837	0.0731
2nd	0.9968	0.9806	0.9831	0.9818	0.0783
3rd	0.9978	0.9814	0.9879	0.9846	0.0755
4th	0.9956	0.9780	0.9897	0.9838	0.0897
5th	0.9939	0.9819	0.9855	0.9837	0.0731
Mean ± standard deviation	0.9963 ± 0.0015	0.9798 ± 0.0021	0.9871 ± 0.0028	0.9834 ± 0.0010	0.0818 ± 0.0088

The experimental results from Tables 8 and 9 indicate that MDDCC has achieved satisfactory outcomes in the detection tests on both the CIC-IDS2017 and CIC-DDoS2019 datasets. The detection accuracy for both datasets surpassed 99.5%, with the recall rates for anomaly samples reaching 99.65% and 98.71% respectively, and the precision rates were also notably high. Additionally, the model exhibited a false positive rate exceeding 8% on the CIC-DDoS2019 dataset. The primary cause of this phenomenon is attributed to the class imbalance within the CIC-DDoS2019 dataset. Due to the relatively smaller number of normal samples, even a small number of misclassifications of normal samples as attack samples can lead to a high FPR.

The detection experiment results of MDDCC on the InSDN, CIC-IDS2017, and CIC-DDoS2019 datasets demonstrate that MDDCC not only has good detection ability on simulated traffic data but also shows excellent detection performance on open network data sets, which shows that the MDDCC model has strong generalization ability.

(d) Performance comparison with other detection methods

To objectively evaluate the performance of the MDDCC model, it was compared with other similar detection models, specifically including CNN-Softmax^19^ and CNN-LSTM^30^, DNN-LSTM^31^, GAN^32^, and 1D-CNN & 2D-CNN^33^, which are traditional classic detection models. Since some literature does not list the false positive rate as a performance metric, only four detection indexes, namely accuracy, accuracy, recall, and F1 value were selected for comparison, with the results shown in Table 10.

**Table 10:** Performance comparison with different detection models.

Model	Dataset	Accuracy	Precision	Recall	F1
CNN-Softmax	InSDN	0.9850	0.9827	0.9827	0.9827
CNN-LSTM	InSDN	0.9632	0.9760	0.9724	0.9742
DNN-LSTM	CIC-IDS2017	0.9932	0.993	0.993	0.993
GAN	CIC-DDoS2019	0.9438	0.9408	0.9789	0.9594
1D-CNN&2D-CNN	CIC-IDS2017	**0.9977**	0.98	0.97	0.98
MDDCC	InSDN	**0.9924**	**0.9968**	**0.9937**	**0.9953**
MDDCC	CIC-IDS2017	0.9959	**0.9985**	**0.9965**	**0.9975**
MDDCC	CIC-DDoS2019	**0.9963**	**0.9798**	**0.9871**	**0.9834**

MDDCC achieved an accuracy of 99.24% on the InSDN dataset, which is an improvement of 0.75% and 3.03% over the accuracies of the CNN-Softmax and CNN-LSTM detection methods, respectively. The precision rate of MDDCC is 99.68%, marking an increase of 1.43% and 2.13% compared to the two methods. The recall rate stands at 99.37%, which is an enhancement of 1.13% and 2.2% respectively. Additionally, the F1 score for MDDCC is 99.53%, showing an improvement of 1.28% and 2.17% when compared to CNN-Softmax and CNN-LSTM. Additionally, on the CIC-IDS2017 dataset, MDDCC achieved an accuracy of 99.59%, which is a 0.27% improvement over the accuracy of DNN-LSTM. Although it is 0.14% lower than the 99.77% accuracy of 1D-CNN & 2D-CNN, MDDCC achieved higher precision and recall rates. Furthermore, MDDCC demonstrated a significant advantage over GAN on the CIC-DDoS2019 dataset. This indicates that the MDDCC model we designed has higher detection accuracy compared to traditional detection models.

#### DDoS attack mitigation test

**Figure 9:**
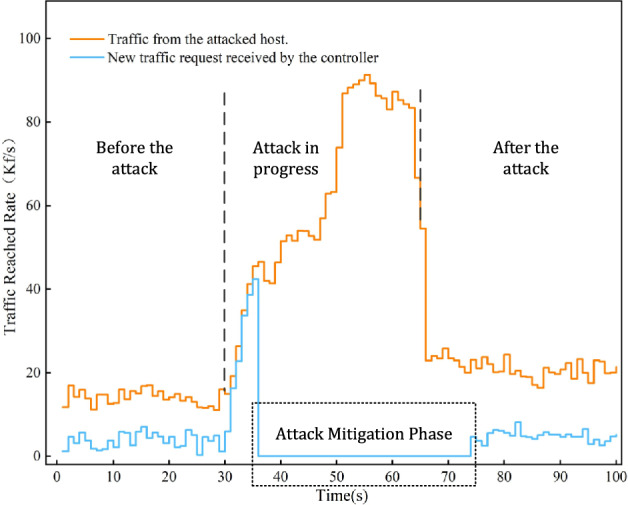
Changes of new flows before and after DDOS attacks and during mitigation.

The attack mitigation experiment uses the new flow rate arriving at the SDN controller (Kf/s, Kilo-flows per second) as the detection metric. Host 1 runs the Hping3 attack program to simulate the DDoS attack source host. At the initiation of the attack, Host 1 sends a large number of packets with random target IP addresses into the SDN network. Once the detection system identifies the network attack, it activates the attack mitigation mechanism to restrict the power of the attacking host to send new flows. Figure 9 illustrates the changes in new flows in the network before and after the DDoS attack, which can be roughly divided into three stages.

- Before the attack is initiated, in the first 30 s, Host 1 continuously sends traffic data containing some new flows into the network at a rate of 20Kf/s. During this phase, the switch forwards the new flow requests from Host 1 to the controller for processing instructions, while other legitimate flows are forwarded normally; hence, there is a certain gap between the number of flows sent by Host 1 and the number of new flows received by the controller.
- During the attack, Host 1 stops sending normal data packets and gradually increases the sending rate of new flows, reaching a maximum rate of 95Kf/s at 55 s, then gradually decreases, and stops the attack at 65 s. In the initial stage of the attack, the switch tries to accommodate the forwarding requests from Host 1, therefore the controller receives a large number of new request packets. However, once the system detects the attack, the mitigation mechanism is activated, and the controller restricts the new flow requests from the attacking host, completely blocking the new flow requests from reaching the controller to prevent the attack from further consuming SDN resources. During this period, the detection system continuously monitors the network status. Additionally, although the attack stops at 65 s, due to the restriction period not being over, it is not until approximately 75 s that Host 1 regains the ability to send new flows, and the controller gradually starts receiving new flow request data again.
- After the attack concludes, Host 1 resumes its data transmission state 65 s later, and the flow rate received by the controller maintains a normal gap with the flow rate sent by Host 1, as it was before the attack.

**Figure 10:**
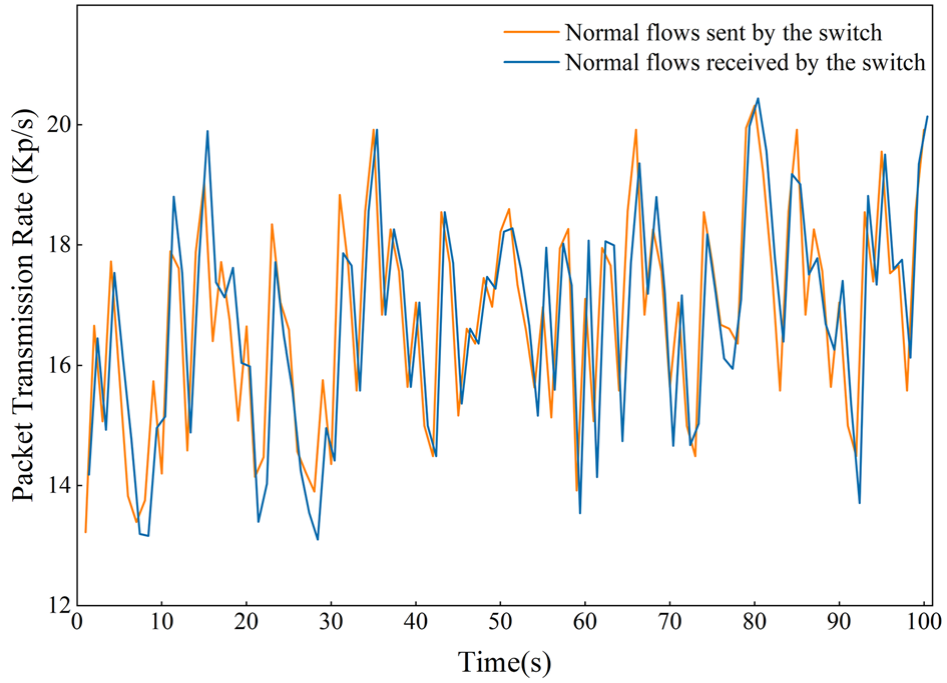
Rate statistics of normal traffic received and sent by switch 1.

Furthermore, during the period when Switch 1 was under attack, Host 2 and Host 3 continued to send traffic data of random intensity as usual. Figure 10 shows the packet reception rate at the port of Switch 1 connected to Host 2 and Host 3, as well as the packet transmission rate from other ports except the controller, with the rates measured in Kilopackets per second (Kp/s). It can be observed that the switch maintains an overall relative balance between receiving and transmitting data packets when forwarding normal data packets, and this balance is consistently maintained even during the initiation and progression of the attack. This indicates that the attack detection and mitigation system we have designed is not only capable of effectively detecting attacks present within the network, but it can also autonomously mitigate the effects of these attacks, ensuring that other hosts in the network can continue to send data normally.

The aforementioned experiments demonstrate that the attack mitigation mechanism we designed can implement restriction strategies on the attack source within a short time after identifying the source of the network attack, thereby ensuring that the SDN has sufficient available resources to provide normal services for the network.

## Conclusion

SDN, as a trend in the development of future networks, urgently requires a fast and efficient anomaly detection method to maintain its own security. We propose a two-stage attack detection approach. First, attack detection based on switch statistics can quickly achieve a coarse-grained detection of network attacks by calculating the statistical information of switch ports, without adding network components and communication volume. Second, attack detection based on multi-dimensional traffic features uses wavelet transform and deep learning technology to perform multi-dimensional and in-depth feature extraction on traffic feature data, which is conducive to accurately classifying traffic samples. Additionally, the traceability method based on graph theory and the identifiers of switches and ports, along with the forwarding restriction-based mitigation strategy, can prevent excessive consumption of SDN resources. The experimental results show that the detection method we proposed can fully utilize the statistical information of switches and the characteristic data of traffic to achieve rapid detection of DDoS attacks and accurate identification of attack samples, achieving higher detection accuracy than traditional methods. Finally, the mitigation mechanism can effectively prevent the SDN controller from being overloaded and maintain the normal operation of the network. Future research will focus on how to apply the proposed method to large-scale SDNs, with an emphasis on addressing the intelligent collaboration issues of multiple controllers in the detection and mitigation process of DDoS attacks.

## Data Availability

The InSDN dataset analysed during the current study is publicly available on https://aseados.ucd.ie/datasets/SDN/InSDN_DatasetCSV.zip. The CIC-IDS2017 and CIC-DDoS2019 datasets are available in the Canadian Institute for Cybersecurity webpage: https://www.unb.ca/cic/datasets/index.htm.

## References

[1] Kreutz D, Ramos FM, Verissimo PE, Rothenberg CE, Azodolmolky S, Uhlig S (2014). Software-defined networking: A comprehensive survey. Proc. IEEE.

[2] El Kamel A, Eltaief H, Youssef H (2022). On-the-fly (D) DoS attack mitigation in SDN using Deep Neural Network-based rate limiting. Comput. Commun..

[3] Wu P, Chang Ch, Zuo ZhB, Ma YY (2022). Address overloading-based packet forwarding verification in SDN. J. Commun..

[4] Fouladi RF, Ermiş O, Anarim E (2022). A DDoS attack detection and countermeasure scheme based on DWT and auto-encoder neural network for SDN. Comput. Netw..

[5] AbdelAzim NM, Fahmy SF, Sobh MA, Eldin AMB (2021). A hybrid entropy-based DoS attacks detection system for software defined networks (SDN): A proposed trust mechanism. Egypt. Inform. J..

[6] Alenezi, F. A., Song, S., & Choi, B. Y. SWANS: SDN-based wormhole analysis using the neighbor similarity for a mobile ad hoc network (MANET). In *2021 IFIP/IEEE International Symposium on Integrated Network Management (IM)*, 653–657 (IEEE, 2021).

[7] Tayfour, O. E., Mubarakali, A., Tayfour, A. E., Marsono, M. N., Hassan, E., & Abdelrahman, A. M. Adapting deep learning-LSTM method using optimized dataset in SDN controller for secure IoT. *Soft Comput.* 1–9 (2023).

[8] Nadeem MW, Goh HG, Ponnusamy V, Aun Y (2022). DDoS detection in SDN using machine learning techniques. Comput. Mater. Continua.

[9] Tang D, Wang X, Yan Y, Zhang D, Zhao H (2022). ADMS: An online attack detection and mitigation system for LDoS attacks via SDN. Comput. Commun..

[10] Sahoo, D., Pham, Q., Lu, J., & Hoi, S. C. Online deep learning: learning deep neural networks on the fly. In *Proceedings of the 27th International Joint Conference on Artificial Intelligence,* 2660–2666 (2018).

[11] Wang S, Balarezo JF, Chavez KG, Al-Hourani A, Kandeepan S, Asghar MR, Russello G (2022). Detecting flooding DDoS attacks in software defined networks using supervised learning techniques. Eng. Sci. Technol. Int. J..

[12] Banitalebi Dehkordi A, Soltanaghaei M, Boroujeni FZ (2021). The DDoS attacks detection through machine learning and statistical methods in SDN. J. Supercomput..

[13] Ali TE, Chong YW, Manickam S (2023). Machine learning techniques to detect a DDoS attack in SDN: A systematic review. Appl. Sci..

[14] Zhou YT, Zhang B, Liu ZH (2022). Application layer DDoS detection model based on multimodal deep learning neural network. Acta Electron. Sin..

[15] Alduailij M, Khan QW, Tahir M, Sardaraz M, Alduailij M, Malik F (2022). Machine-learning-based DDoS attack detection using mutual information and random forest feature importance method. Symmetry.

[16] Zhiyong L, Yu ZH, Qing W, Weiwei S (2023). Study of SDN intrusion intent identification algorithm based on Bayesian attack graph. J. Commun..

[17] Santos R, Souza D, Santo W, Ribeiro A, Moreno E (2020). Machine learning algorithms to detect DDoS attacks in SDN. Concurr. Comput. Pract. Exp..

[18] Elsayed, M. S., Le-Khac, N. A., Dev, S., & Jurcut, A. D. Machine-learning techniques for detecting attacks in SDN. In *2019 IEEE 7th International Conference on Computer Science and Network Technology (ICCSNT)*, 277–281 (IEEE, 2019).

[19] ElSayed MS, Le-Khac NA, Albahar MA, Jurcut A (2021). A novel hybrid model for intrusion detection systems in SDNs based on CNN and a new regularization technique. J. Netw. Comput. Appl..

[20] Gadze JD, Bamfo-Asante AA, Agyemang JO, Nunoo-Mensah H, Opare KAB (2021). An investigation into the application of deep learning in the detection and mitigation of DDOS attack on SDN controllers. Technologies.

[21] Kachavimath, A. V., & Narayan, D. G. Distributed denial of service attacks detection using deep learning in software defined network. In *2022 13th International Conference on Computing Communication and Networking Technologies (ICCCNT)*, 1–5 (IEEE, 2022).

[22] Elsayed MS, Le-Khac NA, Jurcut AD (2020). InSDN: A novel SDN intrusion dataset. IEEE Access.

[23] Lee, T. H., Chang, L. H., & Syu, C. W. Deep learning enabled intrusion detection and prevention system over SDN networks. In *2020 IEEE International Conference on Communications Workshops (ICC Workshops)*, 1–6 (IEEE, 2020).

[24] Yungaicela-Naula NM, Vargas-Rosales C, Pérez-Díaz JA, Carrera DF (2022). A flexible SDN-based framework for slow-rate DDoS attack mitigation by using deep reinforcement learning. J. Netw. Comput. Appl..

[25] Cao Y, Jiang H, Deng Y, Wu J, Zhou P, Luo W (2021). Detecting and mitigating DDoS attacks in SDN using spatial-temporal graph convolutional network. IEEE Trans. Depend. Secure Comput..

[26] Sudar, K. M., & Deepalakshmi, P. Flow-based detection and mitigation of low-rate ddos attack in sdn environment using machine learning techniques. In *IoT and Analytics for Sensor Networks: Proceedings of ICWSNUCA 2021,* 193–205 (Springer Singapore, 2022).

[27] Filali, A., Kobbane, A., Elmachkour, M., & Cherkaoui, S. SDN controller assignment and load balancing with minimum quota of processing capacity. In *2018 IEEE International Conference on Communications (ICC)*, 1–6 (IEEE, 2018).

[28] Yue M, Wang HY, Wu ZJ, Liu L (2020). A survey of DDoS attack and defense technologies in cloud computing. Chin. J. Comput..

[29] Krishnan P, Duttagupta S, Achuthan K (2019). VARMAN: Multi-plane security framework for software defined networks. Comput. Commun..

[30] Elsayed, M. S., Le-Khac, N. A., Jahromi, H. Z., & Jurcut, A. D. A hybrid CNN-LSTM based approach for anomaly detection systems in SDNs. In *Proceedings of the 16th International Conference on Availability, Reliability and Security, Vienna, Austria*, 17–20 (2021).

[31] Tayfour OE, Mubarakali A, Tayfour AE, Marsono MN, Hassan E, Abdelrahman AM (2023). Adapting deep learning-LSTM method using optimized dataset in SDN controller for secure IoT. Soft Comput..

[32] Novaes MP, Carvalho LF, Lloret J, Proença ML (2021). Adversarial deep learning approach detection and defense against DDoS attacks in SDN environments. Future Gener. Comput. Syst..

[33] Alanazi F, Jambi K, Eassa F, Khemakhem M, Basuhail A, Alsubhi K (2022). Ensemble deep learning models for mitigating DDoS attack in software-defined network. Intell. Autom. Soft Comput..

